# Assessment of TSPAN Expression Profile and Their Role in the VSCC Prognosis

**DOI:** 10.3390/ijms22095015

**Published:** 2021-05-09

**Authors:** Kelly Pedrozo Ferreira, Bruna Cristine de Almeida, Laura Gonzalez dos Anjos, Glauco Baiocchi, Fernando Augusto Soares, Rafael Malagoli Rocha, Edmund Chada Baracat, Andrey Senos Dobroff, Katia Candido Carvalho

**Affiliations:** 1 Laboratório de Ginecologia Estrutural e Molecular (LIM 58), Disciplina de Ginecologia, Departamento de Obstetricia e Ginecologia, Hospital das Clinicas da Faculdade de Medicina da Universidade de Sao Paulo, HCFMUSP, SP, BR Av. Dr Arnaldo 455, sala 4121, Cerqueira Cesar, São Paulo 05403-010, Brazil; keeh.pedroz1@gmail.com (K.P.F.); bruc_10@hotmail.com (B.C.d.A.); lauragonzalezanjos@gmail.com (L.G.d.A.); edmund.baracat@hc.fm.usp.br (E.C.B.); 2 Department of Gynecology Oncology, A. C. Camargo Cancer Center, Rua Prof Antonio Prudente 211, São Paulo 01509-001, Brazil; glauco.baiocchi@accamargo.org.br; 3 Department of Pathology, Rede D’OR-São Luiz, Rua das Perobas, 344-Jabaquara, São Paulo 04321-120, Brazil; fasoares@me.com; 4 Department of Gynecology, Federal University of São Paulo (UNIFESP), R. Napoleão de Barros, 608, Vila Clementino, São Paulo 04024-002, Brazil; rafael.malagoli@gmail.com; 5 University of New Mexico Comprehensive Cancer Center (UNMCCC), 1 University of New Mexico, Albuquerque, NM 87131, USA; adobroff@salud.unm.edu; 6 Division of Molecular Medicine, Department of Internal Medicine, University of New Mexico (UNM) School of Medicine, UNM Health Sciences Center, 1 University of New Mexico, MSC07 4025, Cancer Research Facility (CRF) 321A, Albuquerque, NM 87131, USA

**Keywords:** tetraspanins, VSCC, immunohistochemistry, HPV infection, prognosis, cell culture, siRNA

## Abstract

The role and prognostic value of tetraspanins (TSPANs) in vulvar squamous cell carcinoma (VSCC) remain poorly understood. We sought to primarily determine, at both the molecular and tissue level, the expression profile of the TSPANs CD9, CD63, CD81, and CD82 in archived VSCC samples (*n* = 117) and further investigate their clinical relevance as prognostic markers. Our studies led us to identify CD63 as the most highly expressed TSPAN, at the gene and protein levels. Multicomparison studies also revealed that the expression of CD9 was associated with tumor size, whereas CD63 upregulation was associated with histological diagnosis and vascular invasion. Moreover, low expression of CD81 and CD82 was associated with worse prognosis. To determine the role of TSPANs in VSCC at the cellular level, we assessed the mRNA levels of CD63 and CD82 in established metastatic (SW962) and non-metastatic (SW954) VSCC human cell lines. *CD82* was found to be downregulated in SW962 cells, thus supporting its metastasis suppressor role. However, *CD63* was significantly upregulated in both cell lines. Silencing of CD63 by siRNA led to a significant decrease in proliferation of both SW954 and SW962. Furthermore, in SW962 particularly, *CD63-siRNA* also remarkably inhibited cell migration. Altogether, our data suggest that the differential expression of TSPANs represents an important feature for prognosis of VSCC patients and indicates that CD63 and CD82 are likely potential therapeutic targets in VSCC.

## 1. Introduction

Vulvar cancer, albeit rare, represents about 4% of all gynecological malignancies. The American Cancer Society (ACS) estimated that, in 2020, more than 6000 new cases of vulvar cancer were diagnosed in the USA alone, with over 1300 patients dying from the disease due to cancer progression [1,2]. Among all types of vulvar malignancies, vulvar squamous cell carcinoma (VSCC) is the most frequent histological subtype (~90%) [1,3,4]. VSCC may arise from two different etiopathogenic pathways: the first is associated with infection by high-risk human papillomavirus (hrHPV) and the second forms independent of HPV [5]. HPV-associated tumors have a basaloid or verrucous morphology and represent almost half of all VSCC cases, often occurring in younger women. The HPV-independent subset, on the other hand, tends to occur in older women (50+ years old) and is frequently associated with either chronic inflammatory skin conditions, such as lichen sclerosus, lichen planus, or differentiated vulvar intraepithelial neoplasia (d-VIN) [6,7,8].

The involvement of lymph nodes (LN) during VSCC progression carries profound prognostic implications and dictates subsequent treatment and therapy decisions. For instance, while the 5-year survival rate for patients with a negative LN is over 80.7%, the presence of four or more positive inguinofemoral nodes significantly reduces their survival rate to 13% and to less than 11% in patients with distant groin LN metastases [3,9,10,11]. Currently, radical vulvectomy with inguinofemoral lymph node dissection is the surgery of choice to treat VSCC. Unfortunately, such an approach is associated with high morbidity. Hence, to improve prognosis and quality of life for VCSS patients, less invasive therapeutic options are urgently needed. In this context, in-depth studies of the molecular mechanisms underlying VSCC development and progression are necessary to define better and more effective therapeutic options [12,13,14,15,16].

Tetraspanins (TSPANs) are a family of conserved transmembrane proteins that connect intracellular and extracellular compartments. TSPANs, such as CD9, CD37, CD63, CD81, CD82, and CD151, serve as membrane scaffolds, bringing together different classes of surface molecules (i.e., cell surface receptors, immunoglobulins, adhesion molecules), generally through the tetraspanin-enriched microdomains (TEMs) [17,18,19]. As “master organizers” of membrane proteins and because of their numerous potential interacting partners, TSPANs participate in a myriad of fundamental physiological and pathophysiological processes [20,21,22]. Furthermore, TSPANs, in a context-depended manner, are able to either play a key role in immunity against infectious diseases or function as facilitators for viruses, such as HPV [23,24,25], HIV [23,26,27], HCV [28], and MERS-coronavirus [29,30], to enter the cells.

In cancer, several studies have proposed a complex dual role for TSPANs, suggesting that their differential and spatiotemporal expression is associated with either the development and progression of different cancer types (e.g., colorectal [31], breast [32], and epithelial ovarian cancer [33], oral [34,35] and esophageal [36] squamous cell carcinomas) or providing better clinical outcomes [35,37]. For instance, while the expression of the TSPAN CD81, also known as TSPAN28, in breast cancer, multiple myeloma, and acute myeloid leukemia is considered a marker for worse prognosis, in patients with gallbladder carcinoma, its expression indicates a favorable outcome [38,39]. Similarly, the CD63, also known as melanoma-associated antigen ME491 or MLA1, is highly expressed at the tissue level in the initial stages of melanoma development but down-regulated in advanced metastatic lesions, suggesting that CD63 is likely a suppressor of tumor progression [40,41]. Recently, Garcia-Mayea et al. (2020) identified TSPAN1 as an important protein involved in the development, progression, and chemoresistance of HNSCC tumors [42]. Furthermore, An et al. (2020) isolated extracellular vesicles (EV) from patients who underwent surgery for SCC of the lung and observed that both low CD63 and EV expression correlated with unfavorable disease-free survival (DFS) [43].

In vulvar tumors, the expression profile of TSPANs remain poorly elucidated, and thus, their role and contribution for VSCC origin and development, as well as their use as potential therapeutic targets, remain unclear. Herein, we sought to determine the gene and protein expression profiles of four major TSPANs (i.e., CD9, CD63, CD81, and CD82) and investigate their correlation with clinical and pathological features in patients with VSCC. Moreover, we also functionally elucidated the role of *CD63* at the cellular level in both metastatic and non-metastatic vulvar cancer human cell lines. Together, our results provide new clinical and functional insights of TSPANs in VCSS and exploit their role as potential new targets for therapy.

## 2. Results

### 2.1. Case Selection and Clinical Features of the Study Population

For this study, 117 archived VSCC samples were selected from a comprehensive set of 300 vulvar cancers collected from patients who underwent treatment at the AC Camargo Cancer Center (São Paulo, Brazil) between 1978 and 2009 (Table 1) [14,44,45,46,47,48,49]. The criteria for patient/tissue selection were stipulated based on well annotated clinicopathological medical records, as well as clinically relevant parameters for diagnosis, treatment, prognosis, and currently known VSCC risk factors (e.g., HPV) [50]. As depicted in Table 1, the median age of the patients selected for our studies was 67.22 years (ranging from 14 to 98 years old), where the median age for patients in the menopause was 50 years (ranging from 35 to 64 years old), in menarche was 14 (ranging from 11 to 19 years old), and in sexarche was 20 (ranging from 15 to 38 years old). Other clinicopathological parameters, such as tumor size, tumor stage, tumor invasion, metastasis, and HPV infection status, were also evaluated and are summarized in Table 1.

During data analysis, we found that 13% of the patients (*n* = 10) out of 79 medical records available received neoadjuvant chemotherapy as part of their care (Table 1). To further evaluate the impact of neoadjuvant chemotherapy on clinicopathologic variables, we performed a series of univariate and multivariate analyses (Table S1). For instance, our analyses indicate that, within the patients receiving neoadjuvant therapy, 5 patients were reported having perineal commitment against 11 patients in the non-treated arm (*p* = 0.015). Still within the non-treated group (*n* = 68), 75% of them showed tumors larger than 6 mm^3^ (*p* = 0.014), with about 86% of those (*n* = 66) presenting deeper lesions when compared to neoadjuvant treated patients (*p* = 0.050). Additionally, nearly 61% of patients (*n* = 40) in the non-treated group had dermis commitment compared to other lesion types within the same group. The frequency of tumor relapse was also higher in patients that did not receive neoadjuvant chemotherapy (78%) as compared to treated individuals (22%) (*p* = 0.010). Surprisingly, 100% of non-treated patients showed infection by high-risk HPV compared to patients receiving chemotherapy (*p* = 0.021).

Furthermore, we found that 60.8% of patients (*n* = 31) within the 51 VSCC cases with clinical annotated status on HPV-infection were positive for the virus (Table 1). Considering HPV positive patients, 80.6% of them (*n* = 25) exhibited high-risk HPV and 9.7% (*n* = 3) showed a low-risk type of HPV. Considering only patients with high-risk HPV, 28% (7 out of 25) showed coinfection with two or three virus isotypes. Additionally, 9.7% (3 out of 31) of HPV positive samples had coinfection with both high and low-risk types (Table S2). Additionally, the HPV 16 type was found predominantly in 58% of HPV-infected patients (18 out of 31), followed by the HPV 33 type, which affected 23% of patients, as shown in Table S3.

### 2.2. TSPANs Expression in Patients’ Samples

**Figure 1:**
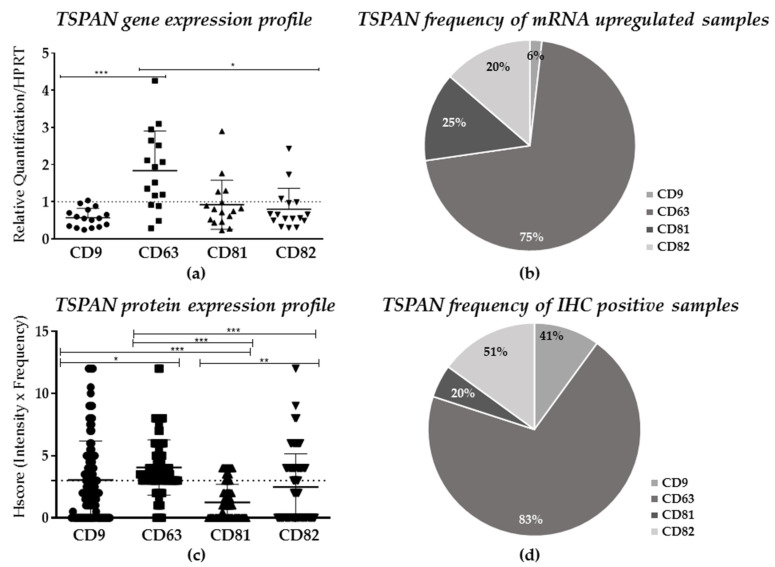
The TSPAN gene and semi-quantitative protein expression profile in VSCC samples. (**a**) Frequency (%) of tissue samples with upregulation (FR > 1) of CD9, CD63, CD81, and CD82 (*n* = 16). (**b**) Relative quantification of TSPAN expression in tumors samples compared to normal, tumor-free edges (tissue reference). Human hypoxanthine phosphoribosyltransferase (HPRT) was used as an internal reference. * *p* = 0.0035; *** *p* = 0.0006. (**c**) Frequency (%) of samples with positive protein expression (*n* = 117). (**d**) Semi-quantitative expression analysis of TSPANs in VSCC samples (* *p* < 0.01; ** *p* < 0.001; *** *p* < 0.0001). Dotted lines indicate the cut off values for RT-PCR (≥1; a) and IHC (≥3, c) analyses.

To determine the expression profile of TSPANs in VSCC at the molecular level, we first performed a quantitative real-time PCR (qRT-PCR) analysis for CD9, CD63, CD81, and CD82 in 16 archived frozen tumors and matched normal adjacent tissues randomly selected from our 117 patients. As depicted in Figure 1a, our analyses revealed that the overall relative expression (mean ± SD) of CD9 was significantly down-regulated by nearly 50% in tumor samples compared to normal tissues (*p* = 0.0006). In contrast, VSCC samples had a significant increase of CD63 mRNA levels by ~1.0-fold (*p* < 0.005), as compared to the control. Notably, no statistical differences were observed when CD81 and CD82 were compared to normal tissues. Upon further investigation of the expression frequency of each TSPAN in our VSCC cohort, we found that CD63 was upregulated in 75% (*n* = 12) of the samples (fold regulation (FR) >1), as compared to its counterparts (CD81 at 25%, CD82 at 20%, and CD9 at 6%, respectively; Figure 1b).

**Figure 2:**
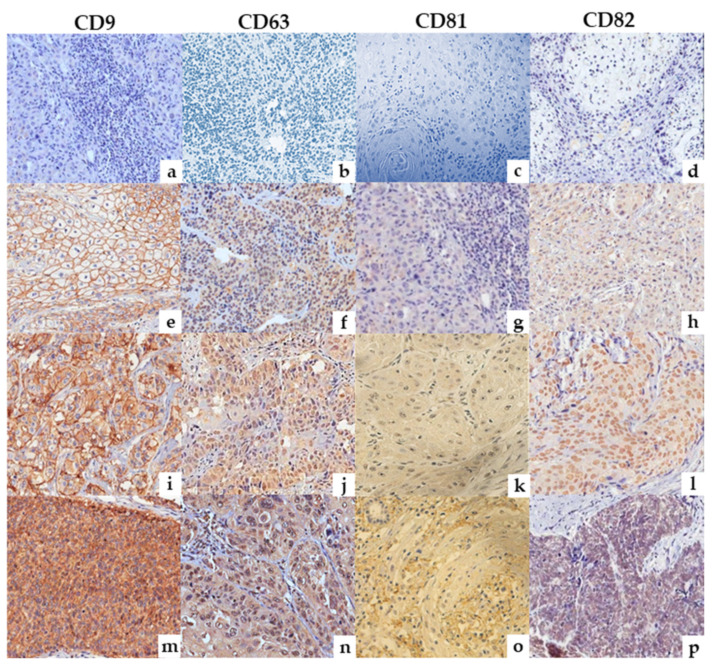
Representative photomicrographs of IHC reactions for CD9, CD63, CD81, and CD82. The pattern of staining was categorized as negative (**a**–**d**), weak (**e**–**h**), moderate (**i**–**l**), and strong (**m**–**p**) reactions (original magnification, ×40), considering membrane and/or cytoplasmic positivity.

Next, to expand on our initial findings and determine whether the protein expression (Figure 1c) and frequency (Figure 1d and Table 2) of each TSPAN were consistent with the gene expression results (Figure 1a,b), a series of immunohistochemical analyses were performed on pre-determined FPPE samples (*n* = 117, Table 1). To ensure reproducibility and consistency in data acquisition and semi-quantitative protein analysis, tonsils were used as a positive control reference (Figures S1 and S2). At the tissue level, CD9 was found to be predominantly localized at the cell membrane, whereas CD63, CD81, and CD82 were primarily found in the cytoplasm of the tumor samples (Figure 2). Our analysis also showed a heterogeneous staining pattern of all four TSPANs, which were classified from a negative to strong expression, regardless of their cell compartment localization, according to the H-score method (0–12). Considering only samples with a H-score result ≥3 (moderated and strong staining—Figure 1c and Figure 2), we found that CD81 was poorly expressed in VSCC samples, with 20% of the tissue showing a dull staining pattern (H-score of ~1.5). CD82 and CD9 were both found to be moderately expressed in most samples with a frequency of 51% and 41%, respectively. Lastly, and similar to the gene expression results, CD63 was found to be more highly expressed (H-score ≥ 4, Figure 1d and Figure 2) and the most frequent (positive in 83% of the samples, Figure 1d and Table 2) TSPAN in all VSCC samples evaluated. Primary antibodies specificity and sensitivity can be seen in Supplementary Figure S3.

Statistically, the comparison analysis within the protein expression of each TSPAN showed significant differences between CD63 and CD9 (*p* < 0.01), as well as between CD9 and CD81 (*p* < 0.0001, Figure 1c). Robust statistical differences were also found among the expressions of CD63, CD81, and CD82 (*p* < 0.0001) and between CD81 and CD82 (*p* < 0.001). Moreover, co-expression analysis among TSPANs showed a moderate positive correlation between CD82 and CD9 (r = 0.597, *p* < 0.0001) and four less robust, yet positive correlations between CD9/CD63 (r = 0.325, *p* = 0.001), CD9/CD81 (r = 0.423, *p* < 0.0001), CD63/CD82 (r = 0.422, *p* < 0.0001), and CD81/CD82 (r = 0.356, *p* < 0.0001) (Table 3), thus suggesting that TSPANs may interact with each other in VSCC.

### 2.3. Clinical Implication of TSPANs Expression for VSCC Patients

Based on the unique molecular and tissue signature of CD9, CD63, CD81, and CD82, we sought to investigate whether their expression could be clinically useful as markers for diagnosis and/or prognosis or used to predict patterns of treatment response and outcome. Therefore, their expression profiles were evaluated in tandem with the patients’ clinical features and available follow-up medical information.

**Figure 3:**
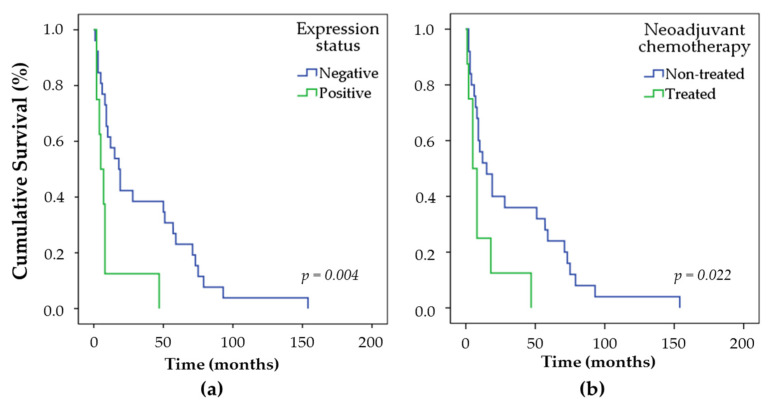
Kaplan–Meier Curves for DFS, according to CD81 status (*n* = 105). (**a**) In total, 77% of the CD81 negative patients showed cancer relapse, while 23% of relapse was recorded among CD81 positive patients (*p* = 0.004). The total number of patients with cancer relapse was 34 (100%). (**b**) Neoadjuvant chemotherapy analysis, including 8 treated and 25 non-treated relapsed VSCC patients (*p* = 0.022) (*n* = 33 *). *p*-value was determined using the log-rank test, which refers to the corresponding expression status. * One patient was excluded due to lack of treatment information.

Although the overall survival (OS) analyses showed no statistical differences for either CD9 (*n* = 79; *p* = 0.950), CD63 (*n* = 76; *p* = 0.485), CD81 (*n* = 76; *p* = 0.205), or CD82 (*n* = 76; *p* = 0.923) expression, disease-free survival (DFS) (Figure 3a) showed that patients with CD81 positive tumors (23%) had a median relapse rate of 5 ± 2.12 months, whereas CD81 negative tumors (77%) showed a median relapse rate of 18 ± 4.46 months (*n* = 34; *p* = 0.004). Further univariate analysis of relapsed patients (CD81+: 8 patients; CD81-: 25 patients) that did or did not receive neoadjuvant chemotherapy regardless of CD81 status revealed that non-treated patients had a better DFS over 25 months (15.00 ± 5.62; 95% CI: 3.98–26.01) than treated patients (5.00 ± 2.12; 95% confidence interval (95% CI): 0.84–9.15) (Figure 3b). Multivariate analysis, considering the expression status of CD81 and neoadjuvant chemotherapy (Cox regression model), showed that CD81 expression is not an independent prognostic factor for VSCC (*p* > 0.05) (Table 4).

Secondary analysis revealed that the CD63 did not show any significant association with clinical features when samples were grouped in positive (Hscore > 3 to 12) or negative (Hscore 0 to 3) groups (Table S5). However, by stratifying the patients into negative/weak, moderate, and strong expressions, we found that moderate expression of CD63 (Hscore ≥ 6 to ≤ 12) strongly correlated with histological diagnosis (*p* < 0.022), treatment (*p* < 0.0162), and vascular invasion (*p* < 0.0009, Table S6). In regard to histological diagnosis, we found that 83% of the VSCC I and 76% of the VSCC II cases were positive for CD63 (*p* < 0.0022, Table S6).

CD81 down-expression was mainly associated with tumor location, treatment, and death. Specifically, 73% of the cases with labia majora commitment were attributed to patients with low CD81 expression, compared to 27% of similar cases in CD81 positive samples (*p* = 0.029). In total, 85% of patients with CD81 negative tumors did not undergo neoadjuvant chemotherapy or radiotherapy (*p* = 0.023), and low expression of CD81 was associated with increasing mortality as compared to CD81 positive patients (*p* = 0.039, Table S7). 

Lastly, we found that the majority of large tumors (>6 mm^3^) were negative for CD82, while in contrast, CD82 positive tumors represented about 61% of smallest tumor samples (<5 mm^3^, *p* = 0.027). A large number of CD82 positive samples was also found to be associated with patients who underwent adjuvant therapy (*p* = 0.004). Moreover, 60% of the CD82 positive tumors show HPV coinfection (*p* = 0.051, Table S8).

No other clinicopathological feature showed relevant statistical significance associated with the expression of TSPANs at the gene or protein levels. A complete list of the variables analyzed is shown in Tables S2–S6.

### 2.4. Role of TSPANs in Metastatic and Non-Metastatic Cell Lines

To mechanistically investigate the role of TSPANs in VSCC, we selected the tetraspanins CD63 and CD82 to study, as they are well characterized as having opposite roles in tumor development and prognosis in the literature.

**Figure 4:**
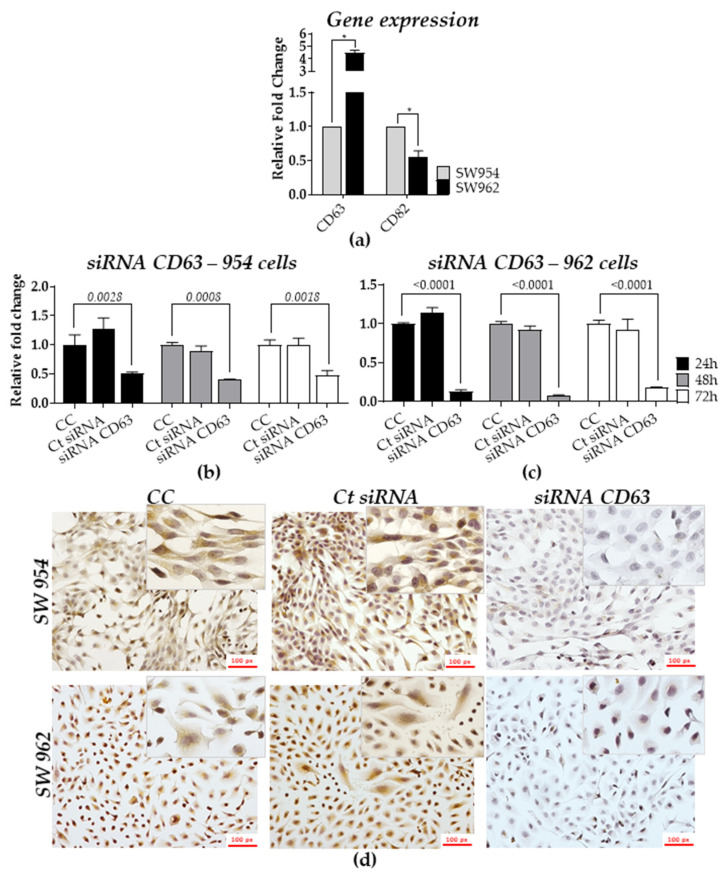
In vitro TSPANs expression in both non-metastatic (SW954) and metastatic (SW962) cell lines. (**a**) Basal transcript expression levels for CD63 and CD82 in SW962 cells, as compared with SW954 (RQ = 1). Expression values were determined after normalization with β2 microglobulin (B2M); relative expression of CD63 in SW954 (**b**) and in SW962 (**c**) cells 24, 48, and 72 h after CD63-siRNA transfection; (**d**) CD63 protein expression assessment by ICC (scale bars, 100 px) 48 h after cells transfection. Results were plotted as mean values ± standard deviation (SD). * *p* < 0.05. CC: Non-transfected cells, Ct siRNA: siRNA control (eGFP-siRNA) and CD63 siRNA: CD63-inhibited cells.

First, we assessed the basal expression profiles of CD63 and CD82 by qRT-PCR in both SW954 (non-metastatic) and SW962 (metastatic) human cell lines. Figure 4a shows that the expression of CD63 was 4.5 times higher in SW962 compared to SW954 cells (*p* < 0.05), whereas the expression of CD82 was decreased by nearly 2 times in the metastatic cells compared to SW954 (*p* < 0.05). 

Next, we transiently silenced CD63 expression by transfecting both cell lines with a small interfering RNA (siRNA). By qRT-PCR, we found that SW954 and SW962 cells transfected with CD63-siRNA had a significant decrease of CD63 mRNA by 70% and 90% (*p* < 0.05), respectively, as compared to the non-transfected cells (CC) and siRNA control (Ct siRNA) (Figure 4b,c). Consistently, at the protein level, the intensity of CD63 staining in immunocytochemistry (ICC) was found to be reduced by 80% and 90% in SW954 and SW962 CD63-siRNA transfected cells, respectively, as compared to the controls (Figure 4d). Further semi-quantitative analysis in both silenced cell lines showed a significant statistical difference in downregulation of CD63 expression (using the score of intensity and frequency of stained cells) in both cell lines when compared to the controls (*p* = 0.0005, Table 5).

**Figure 5:**
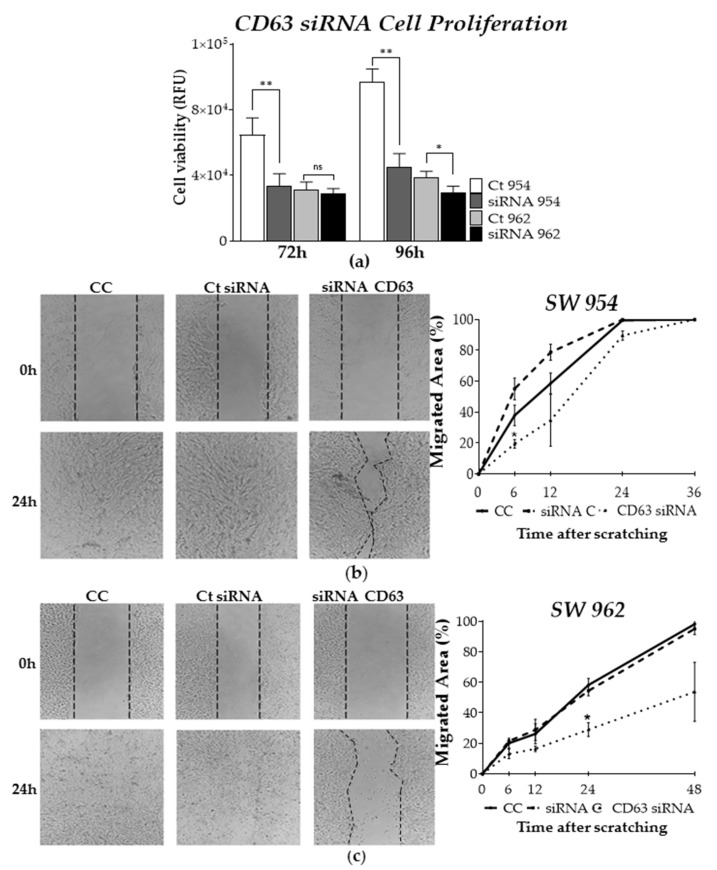
Effects of CD63 silencing on SW954 and SW962 cell proliferation (viability) and migration. (**a**) Cell viability measurements of CD63-siRNA transfected and control cells at different time points (* *p* < 0.05; ** *p* = 0.0022; ns = not significant). Silencing of CD63 in SW954 (**b**) and SW962 (**c**) cells inhibits their motility, as determined by the scratch wound healing assay at 6 h (*p* < 0.05) and 24 h (*p* < 0.05) after scratch, respectively. Representative photomicrographs (original magnification ×10) of scratched wells at time 0 h and 24 h are shown. Experiments with both cells were carried out using siRNA-treated cells for 48 h, and cell migration analysis was carried out until total wound healing of control groups (36 h and 48 h for 954 and 962 cells, respectively). Data are shown as mean values ± standard deviation (± SD) or percentage (%). CC: Non-transfected cells, siRNA C: siRNA control (eGFP-siRNA) and CD63: CD63-siRNA.

At the cellular level, silencing of CD63 in both cell lines led to a significant reduction in cell viability (proliferation assay) as early as 24 h and up to 96 h after *CD63-siRNA* transfection. Based on the siRNA efficiency data, we assessed the CD63 silencing effects 48 h after transfection. Significant differences in the cell proliferation were observed for *CD63-siRNA* SW954 cells after 72 h (~50% of inhibition, *p* < 0.05) and 96 h (~70% of inhibition, *p* < 0.005) (Figure 5a), while *CD63-siRNA* SW962 cells showed higher inhibition rates after 96 h (~20%, *p* < 0.05) (Figure 5a). CD63-dependent cell migration was also investigated in both siRNA transfected and controls cells using the wound healing (scratch) assay 48 h after *CD63* silencing. As depicted in Figure 5b, the ability migration of SW954 cells was compromised after CD63 silencing. However, a significant effect was observed 6 h after the scratch (*CC* = 40%, *siRNA C* = 55%, *CD63-siRNA* = 20%; *p* < 0.05, Figure 5b), and after 12 h, the effect was sustained but without statistical significance. Similarly, a robust and pronounced migration inhibition was also observed in CD63-silenced SW962 cells 24 h after scratch (*CC* = 50%, *siRNA C* = 50%, *CD63-siRNA* = 25%; *p* < 0.05, Figure 5c), without wound closure even after 48 h (*CC* = 90%, *siRNA C* = 90%, *CD63-siRNA* = 50%; *p* > 0.05). The migration analysis of the cells has continued until total wound healing in the control cells group. 

**Figure 6:**
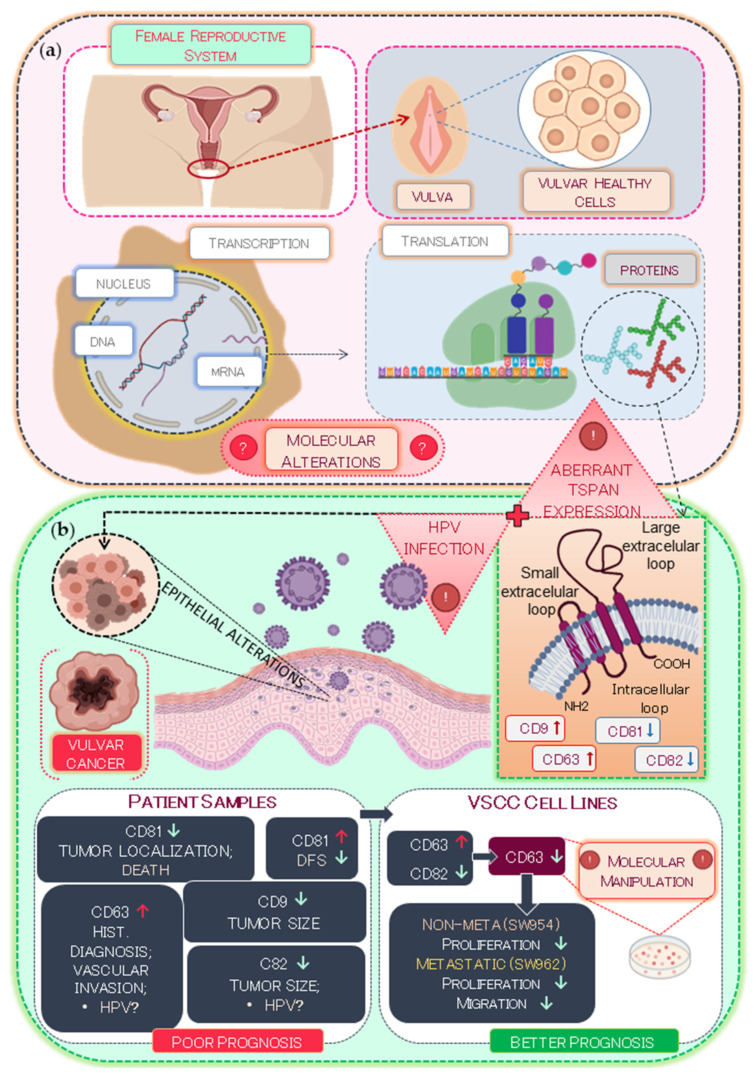
Schematic illustration of TSPAN expression and their prognostic value for VSCC patients. (**a**) During the TSPAN transcription and translation processes, genetic or epigenetic alterations may occur at the molecular level, leading to loss or gain of expression of certain TSPANs that can contribute to several pathogenic processes. (**b**) These alterations, together with high-risk HPV infections, may be associated with larger tumor size, lymph node metastasis, and lower DFS rates, which represent a poor prognosis for VSCC patients. CD63 is related to increased cell survival and migration in the VSCC in vitro analyses. Red and green arrows indicate upregulation and downregulation of genes.

Based on our findings, we propose, as schematically represented in Figure 6, that the differential expression of TSPANs, in particular CD9, CD63, CD81, and CD82, might contribute to VSCC development and poor prognosis. Our results indicate that these alterations, along with high risk-HPV infections, are associated mainly with larger tumor size, lymph node metastasis, and mortality. In vitro, we showed that the expression of CD63 is associated with increased cell survival and migration in the VSCC cells, highlighting the potential clinical benefit of targeting CD63 as a novel therapeutic modality for patients with VSCC.

## 3. Discussion

In the present study, 117 FFPE archived samples of VSCC (from 1978 to 2009) and associated medical records were selected from a larger cohort of 300 samples previously and exhaustively characterized by several studies [14,44,45,46,47,48,49]. A review study published by Maia et al. in 2012 highlighted the uniqueness of the Brazilian’s VSCC registry and concluded that the main prognostic factors for VSCC are mainly the patient’s age, treatment, recurrence, and survival, corroborated with the global literature at the time. Nearly a decade later, the main prognostic factors for VSCC remain virtually unchanged and features such as the patient’s age, HPV/16 status, VINs occurrence, tumor stage and grade, tumor size, depth of invasion, lymph node metastasis, stromal change, and margin status continue to be relevant [1,7,9,10,16,51,52]. As such, our analysis revealed that the patient’s age (~70 years old), therapy status (~80% of patients did not receive any therapy), VSCC stage (~87% of the tumors were VSCC I/II), FIGO stage (~25% of patients were stage IIIB), tumor recurrence (~50% of patients had recurrence), LN metastasis (~40% of patients did present LN involvement), and mortality rate (~54% of the patients died) were adequate prognostic features for selection of the study population and further correlative studies.

Although prognostic factors, such as those discussed above, are clinically important for patient management and decision making, the lack of associated molecular markers to further predict patient outcome (e, OS, DFS, and objective response rate) or to anticipate VSCC progression remain an unmet clinical need. It is well stablished in the literature that alterations in TSPAN expression are directly correlated with prognosis in many cancer types [1]. However, in VSCC, their expression profile and molecular role remain poorly elucidated. Here, we found that, among all four TSPANs analyzed, CD63 was predominantly expressed and more frequent in VSCC samples at the molecular (qRT-PCR, 75%) and tissue level (IHC, 83%) than CD9, CD81, and CD82. Curiously, no significant differences at OS were found when the expression of CD63 or the other three TSPANs were evaluated in conjunction with the patient’s clinical record.

CD9 is ubiquitously expressed in various normal and cancer tissues [53,54], and consequently, its expression as a molecular marker is often ambiguous and cancer-dependent [36,55,56,57,58,59,60,61,62]. Higher positive expression of CD9 has served as a good prognostic marker in breast, lung, colon, and pancreatic carcinomas [55,63,64], as it is thought to have a metastatic suppressor role by inhibiting tumor cell motility, at least in vitro [65,66]. However, in a previous study of tumor progression in penile squamous cell carcinomas [67], we observed that invasive tumors have a heterogeneous pattern of CD9 expression, ranging from strong expression in 80% of the cells to a marked loss of CD9 expression in the same case. Similarly, Hori et al. (2004) and Soyuer et al. (2010) described that higher CD9 expression was associated with more aggressive tumors and increased number of lymph node metastases in gastric cancers, therefore representing a marker of poor prognosis [60,61]. In VSCC, although a direct correlation between CD9 expression and the potential of lymph node metastasis and survival was not statistically significant, we anticipate that patients that are CD9-low or CD9-negative will, more likely, bear tumors that are phenotypically more aggressive and, therefore, have a worse prognosis.

Likewise, the low expression of CD82 in the tissue samples analyzed was associated with larger tumors, as well as increased stromal invasion. Such findings are consistent with previous studies, where downregulation of CD82 expression was associated with both advanced cancer stages (e.g., in breast, bladder, ovary, and prostate carcinomas and also in oral/esophageal/laryngeal/penile squamous cell carcinomas) and the transition to a metastatic phenotype [37,59,68,69,70]. For example, Yang et al. (2001) reported that the overexpression of CD82 in breast cancer cells resulted in the suppression of in vitro invasion and in vivo metastasis, suggesting that CD82 does function as a tumor suppressor [71]. While the tumor suppressing ability of CD82 was also shown in bladder, oral cancer, prostate, and ovary cancers, where cancer cell migration and invasion were drastically reduced upon its upregulation [72,73,74,75,76], in penile cancer, loss of CD82 led to an increase in tumor metastasis and higher HPV16 infection rates [24]. In this context, our analysis did reveal that a large majority of CD82 negative tumors (61% of 30 patients) were associated with HPV16, thus further indicating that the lack of CD82 expression may contribute to a poor prognosis in vulvar carcinomas. The etiological role of HPV in the development of vulvar cancer has been well recognized [77], and further elucidating the role of TSPANs in this process may be a key point for better understanding VSCC development. 

On the other hand, both CD63 and CD81 were found to be overexpressed among the VSCC samples analyzed. Our results showed that the strong CD63 tissue staining was particularly associated with well-differentiated VSCC1 + VSCC2 samples, while lost or downregulated in more aggressive tumors. A similar correlation between high expression of CD63 and larger tumor size, as well as increased vascular and stromal invasion, and higher mortality was observed in our analysis. We found that these results are well aligned with recent publications, suggesting that increased expression of CD63 in certain cancer is correlated with increased activity of pro-tumorigenic cell signaling pathways, such as activation of β-catenin, phosphoinositide 3-kinases (PI3K), and extracellular signal-regulated kinases (ERK) [5]. Furthermore, its increased expression was also reported as an indicator of poor disease specific survival (DSS) in solid tumors [3,68], thus corroborating our clinical findings and suggesting that CD63 functions as an oncogene in VSCC. Indeed, our in vitro analyses showed that silencing of CD63 reduced the proliferation rate of both VSCC cell lines and the migration capacity of the metastatic cells.

Lastly, the expression of CD81 in VSCC samples was robustly correlated with tumor relapse, especially in patients who underwent neoadjuvant therapy. In fact, in the latter, we found a staggering tumor relapse median that was three times higher than untreated patients. Such observation can be attributed to the fact the that CD81 has been shown to regulate cell migration and invasion and, therefore, is implicated in cancer progression and chemoresistance. As such, its overexpression or downregulation has served as a prognostic marker in several tumor types, including breast, lung, prostate, melanoma, brain cancer, and lymphoma [78,79,80]. In our analysis, despite the low frequency of CD81 positive cases, the results indicate that CD81 contributes to a worse DFS and unfavorable prognosis, further corroborating with the literature [81,82]. 

Although more comprehensive analyses are necessary to better understand the role of TSPAN in VSCC, our data indicate that these molecules are important benchmarks of VSCC development and progression. Furthermore, to the best of our knowledge, this was the first study of its kind shedding some light onto the relationship between TSPANs and VSCC, and as such, we believe this work will pave the way for the development of novel treatment modalities, as well as new perspectives for disease management options.

## 4. Materials and Methods

### 4.1. Patient Samples 

The present study was approved by the Research Ethics Committee of the Faculdade de Medicina da Universidade de Sao Paulo-FMUSP (Number 1.540.225) and complied with the Helsinki Declaration. Selected samples included 117 formalin-fixed paraffin-embedded (FFPE) tissues with invasive VSCC diagnoses, which were collected between 1979 and 2009 at the Anatomic Pathology Department of the AC Camargo Cancer Center in Sao Paulo, Brazil [15]. A total of 16 frozen tissues were further selected from the 117 cohort, along with 16 donor-matching morphologically normal tissue edges, for transcriptional analysis of the TSPANs.

### 4.2. Cell Lines, Culture Conditions, and Authentication

Non-metastatic-SW954 (American Type Culture Collection [ATCC] HTB-117) and metastatic-SW962 (ATCC HTB-118) VSCC human cell lines were grown in Roswell Park Memorial Institute-1640 (RPMI; Vitrocell, Embriolife, Sao Paulo, SP, Brazil), medium supplemented with 2 g/L of sodium bicarbonate (Sigma, Darmstadt, HE, Germany), 10% (*v/v*) fetal bovine serum (FBS; Invitrogen, Carlsbad, CA, USA), and antibiotic mix (ampicillin/streptomycin/amphotericin; Gibco, Waltham, MA, USA). Cells were cultured in 75 cm^2^ flasks and maintained at 37 °C in a humidified atmosphere containing 5% CO_2_. Cell line authentication was performed using GenePrint 10 System (Promega, Madison, WI, USA), followed by identification and matching of the short tandem repeat profiles to cell line profiles at the ATCC data bank [83].

### 4.3. Total RNA Isolation, Complementary DNA (cDNA) Synthesis, and Quantitative Real-Time PCR (qRT-PCR)

Total RNA was extracted from frozen tissues using the RNeasy Mini Kit (Qiagen, Hilden, Germany), following the manufacturer’s recommendations. RNA isolation from cell lines (SW954 and SW962) was performed with Trizol (Thermo Fisher Scientific, Carlsbad, CA, USA), according to the manufacturer’s instructions. For studies using TSPAN-silenced cells, 5 × 10^6^ SW954 and SW962 cells were seeded in 4-well plates (NUNC™ Delta Treated, Thermo Fisher Scientific, Carlsbad, CA, USA) and transfected with specific TSPANs, targeting siRNA or siRNA control (described in detail below). Total RNA from silenced cells was isolated using the PureLink RNA Mini Kit (Ambion, Austin, TX, USA), following the manufacturer’s protocol. All RNA samples were quantified with spectrophotometer NanoDrop 2000 (Thermo Fisher Scientific, Carlsbad, CA, USA) prior to downstream applications. cDNA synthesis (reverse transcription) was carried out using the High-Capacity cDNA Reverse Transcription Kit (Thermo Fisher Scientific, Carlsbad, CA, USA). To evaluate the expression of each TSPAN, TaqMan assays for *CD9* (assay ID: Hs00233521_m1), *CD63* (assay ID: Hs00156390_m1), *CD81* (assay ID: Hs01002167_m1), and *CD82* (assay ID: Hs00356310_m1) were used, following the manufacturer’s recommendations. B2M (TaqMan assay ID: Hs99999907_m1) and HRPT (TaqMan assay ID: Hs99999909_m1) were predicted as the stable normalizer by the NormFinder algorithm software [84] and used in our studies as housekeeping (HK) genes for data normalization. qRT-PCR amplifications were performed in an ABI 7500 Real-Time PCR instrument (Thermo Fisher Scientific, Carlsbad, CA, USA). As a reference sample, a pool of RNA isolated from tumor-free edge tissues (*n* = 16) was used. Amplifications were run in duplicate, and averages were obtained after normalization with either *B2M* (for cells) or *HPRT* (for tissues). Relative quantification was set up comparing cycle threshold by ΔΔCT method in SDS 3.0 and RQ manager LV2.0.6 software, and the fold regulation (FR) expression cut-off values were established in ≥+2 and ≤−2 to identify differentially expressed genes.

### 4.4. TMA and Immunohistochemistry (IHC) Analysis

TMA construction was performed in duplicate with selected tumor blocks and adjacent normal tissue samples using the manual arraying instrument (Manual Tissue Arrayer 1, Beecher Instruments Microarray Technology, Sun Prairie, MI, USA). Cylinders of 2 mm diameter were punched from distinct selected areas of each donor paraffin block, as previously described [15]. IHC reactions were carried out with CD9 (Neomarkers, Portsmouth, NH, USA; dilution 1:300), CD63 (Neomarkers, Portsmouth, NH, USA; dilution 1:600), CD81 (Novocastra Laboratories, Benton Ln, Newcastle, UK; dilution 1:30), and CD82 (Neomarkers, Portsmouth, NH, USA; dilution 1:100) antibodies. Sections were blocked with protein block serum-free (Dako, Carpinteria, CA, USA) at room temperature for 20 min. Incubations with primary antibodies were performed at room temperature for 2 h, followed by incubation with an indirect dextran polymer detection system for 1 h (Novocastra Laboratories, Newcastle, UK). After several washes, slides were incubated with 3,3-diaminobenzidine tetrahydrochloride (DAB) (Dako, Carpinteria, CA, USA) for staining. All reactions were run in quadruplicate (two cores per slide, with two stained slides).

Two different pathologists performed semi-quantitative analysis, evaluating the average area of slides staining. Samples were scored using the intensity of staining and frequency of positive tumor cells, as established elsewhere [59,85]. Staining intensity was categorized into four groups: 0 (negative), 1 (weak), 2 (moderate), and 3 (strong), and the frequency score of stained tumor cells was ranked into: 1 (less than 10%), 2 (10 to 50%), 3 (50 to 75%), and 4 (more than 75%). A combined score using the intensity and frequency multiplication was then defined, ranging from 0 to 12, generating four groups: negative (score 0–3), weakly positive (score 4–5), moderate (score 6–8), and strongly positive (9–12). For statistical analysis, only two categorized groups of negative (score 0–3) and positive (score 4–12) expression were used. Normal human tonsil samples were used as a positive control tissue, as recommended by the manufacturer. For negative controls, tissues were stained without primary antibodies (Figure S2). 

### 4.5. DNA Isolation and HPV Genotyping

Total DNA was extracted from up to eight tissue sections (10-μm-thick). Sections were pretreated with 350 μL ATL lysis buffer from the DNA FFPE kit (Qiagen, Valencia, CA, USA) and added directly to the paraffin sections, followed by incubation of the tightly closed tubes at 120 °C for 20 min. All procedures followed the supplier’s specification. DNA was then quantified at the NanoDrop ND-1000 spectrophotometer (Wilmington, DE, USA) and analyzed on 1% agarose gel. 

HPV detection and typing was performed using the linear array HPV-genotyping test (Roche Molecular Systems; Branchburg, NJ, USA). The assay was based on L1 consensus PCR with PGMY primers, yielding a 450-bp amplicon, with type-specific hybridization to detect 37 individual types (6, 11, 16, 18, 26, 31, 33, 35, 39, 40, 42, 45, 51, 52, 53, 54, 55, 56, 58, 59, 61, 62, 64, 66, 67, 68, 69, 70, 71, 72 73, 81, 82, 83, 84, 89, and IS39). In addition, the assay included β-globin primers (150-bp amplicon) detected on the HPV typing strip as a positive control for amplifiable sample DNA. Linear array HPV-genotyping strips were manually interpreted using the HPV reference guide provided. The products of hybridization were detected by a color reaction with an alkaline phosphatase–streptavidin conjugate and substrate (5-bromo-4-chloro-3-indolyl phosphate and nitrobluetetrazolium), which results in a purple precipitate. Hybridization results were visually assessed by comparison with the standard grid [86]. 

### 4.6. CD63 Silencing-siRNA Transfection

Silencing of CD63 was performed using MISSION siRNA *CD63* (EHU032781—Sigma-Aldrich, MO, USA). Cells were cultured in Opti-MEM I Reduced Serum Medium (Thermo Fisher Scientific, Carlsbad, CA, USA), and the siRNA transfection was carried out with Lipofectamine RNAiMAX Reagent (Invitrogen, CA, USA) without antibiotic, according to manufacturer’s instructions. The siRNA eGFP (EHUEGFP—Sigma-Aldrich, Saint-Louis, MO, USA) was used as a negative control. Cell seeding density and the concentration of each reagent were standardized for each functional assay, according to the manufacturer’s recommendations.

### 4.7. Immunocytochemistry (ICC)

Prior to ICC studies, the specificity of the anti-CD63 antibody (MA1-19281, Thermo Fisher Scientific, Carlsbad, CA, USA, Figure S3) was validated and confirmed by western blot analysis (Figure S3). Because western blot analysis was not feasible due to the small number of cells recovered after siRNA transfection, ICC was the method of choice to assess the expression of CD63. All reactions were carried out in 4-well plates (NUNC™ Delta Treated, Thermo Fisher Scientific, GE, USA), with 10^5^ cells/well. Plates were washed with phosphate buffered saline (PBS), and 500 µL of 3.7% formaldehyde (Merck, Darmstadt, HE, Germany) was added for cell fixation. Hydrogen peroxide (H_2_O_2_; VIC pharma, Sao Paulo, BR) was applied to block the endogenous peroxidase and nonspecific proteins. Next, cells were incubated with PBS and 5% bovine serum albumin (BSA) for 20 min at room temperature. For permeabilization, 0.1% Triton X 100 (Merck, Darmstadt, HE, Germany) in PBS was added for 20 min. Anti-CD63 antibody was diluted 1:100 in PBS supplemented with 0.1% Triton X 100 and incubated at room temperature for 1 h. Cells were submitted to three washing steps and incubated with biotinylated secondary antibody for 20 min at room temperature. After several washes, polymer HRP-peroxidase was added and extensively washed. Samples were stained with DAB (1:50, Merck, Darmstadt, HE, Germany) and counterstained with hematoxylin for 10 min at room temperature. Microphotographs and analysis were carried out with optical microscope Nikon Fase Contrast 0.90 Dry-Eclipse Ni (Nikon, Tokyo, Japan), equipped with a photo camera Nikon DS-Ri1 (Nikon, Tokyo, Japan).

### 4.8. Cell Viability Assay

CD63-silenced SW954 (3.4 × 10^4^/well) and SW962 (2 × 10^4^/well) and controls cells were seeded into 96-well treated microplates (Corning, Glendale, AZ, USA) under normal growth conditions. Cell viability was measured over time with PrestoBlue Cell Viability Reagent for Microplates (ThermoFisher Scientific, Carlsbad, CA, USA) (10:100 dilution), according to the manufacturer’s protocol. Fluorescence was measured by a GloMax-Multi Detection System (Promega, Madison, WI, USA).

### 4.9. Wound Healing Assay

SW954 (2 × 10^5^/well) and SW962 (1 × 10^5^/well) cells were seeded in 24-well plates and allowed to reach complete confluence in growth media containing no antibiotics. To make the wound, a plastic P200 pipette tip (Fisher) was used to scratch the cell monolayer to create a cleared area. The wounded cell layer was washed once with 1X PBS to remove loose cells and then transfected with CD63-siRNA and refed with fresh growth media containing 1% FBS. The wounds were observed using phase contrast microscopy on an inverted microscope Zeiss (AxioCam ERc 5c, Wetzlar, HE, Germany). Images were taken at regular intervals over the course of 0 to 48 h and then analyzed by ImageJ (v1.50i, Bethesda, MD, USA).

### 4.10. Statistical Analysis

Distribution of continuous data was analyzed by the Shapiro–Wilk normality test. For comparison between TSPAN expression profiles in SW954 and SW962 cells, the Mann–Whitney U test was used. The Kruskal–Wallis with Dunn’s post-test was used to compare the expression levels of different TSPANs. To evaluate the statistical differences among frequencies of each variable, the Chi-square test or Fisher’s exact test was used. The mean and SD (mean ± SD) were used to represent the patient’s age, as previously described [87]. The Kaplan–Meier method evaluated patient outcome, and the differences between each group were analyzed using the log-rank test (Mantel-Cox) and multivariate analysis with Cox’s regression test by the Cox proportional hazard model. Survival rates were calculated based on months. OS time was set up between the date of the patient’s surgery and either death date or the last information date/follow-up. DFS was determined from the date of surgery to the date of relapse or the last follow-up. Spearman’s rank correlation coefficient was used to measure the strength of the linear relationship between two non-parametric variables, as described elsewhere [87,88]. Statistical analysis was performed using the SPSS (v21, IBM Corp., Armonk, NY, USA) and Graph Pad Prism 5.0 (v3, GraphPad Software Inc., San Diego, CA, USA) software [87]. All differences were considered statistically significant when *p* < 0.05.

## 5. Conclusions

We saw that the imbalance in the TSPAN expression is associated with poor prognosis in VSCC patients. CD63, the most frequent protein found in the samples, seems to have an important and possibly stage-specific role in those tumors. Additionally, CD82 and CD63 might be potential targets for therapy in VSCC.

## Figures and Tables

**Table 1:** Clinical and histopathological features of patients with VSCC (*n* = 117).

Variables	Categories	Overall Population by Medical Record/Percentage Frequencies
**Age**	≤50	12 (10.5%)
	>50	102 (89.5%)
**Menarche**	11 to 14 years old	41 (68.3%)
	15 to 19 years old	19 (31.7%)
**Menopause**	No	3 (4.6%)
	Yes	62 (95.4%)
**Sexarche**	15 to 18 years old	16 (51.6%)
	≥19 years old	15 (48.4%)
**Number of partners**	≤2	36 (92.3%)
	≥3	3 (7.7%)
**Symptoms**	No	1 (1.2%)
	Yes	86 (98.8%)
**Contraceptive**	No	61 (92.4%)
	Yes	5 (7.6%)
**Alcoholism**	No	74 (98.6%)
	Yes	1 (1.4%)
**Smoking**	No	59 (77%)
	Yes	17 (23%)
**Tumor size (mm^3^)**	≤5 mm	42 (37.5%)
	6 to 10 mm	21 (18.8%)
	≥10 mm	49 (43.7%)
**Histological diagnosis**	VSCC 1	44 (38%)
	VSCC 2	47 (40.5%)
	VSCC 3	11 (9.5%)
	Basaloid carcinoma	11 (9.5%)
	Verrucous carcinoma	3 (2.5%)
**Neoadjuvant chemotherapy**	No	69 (87%)
	Yes	10 (13%)
**Adjuvant chemotherapy**	No	76 (91.5%)
	Yes	7 (8.5%)
**Adjuvant radiotherapy**	No	73 (89%)
	Yes	9 (11%)
**Pelvic lymphadenectomy**	No	69 (89.6%)
	Yes	8 (10.4%)
**Depth of invasion**	Superficial dermis	11 (9.8%)
	Dermis	70 (62.5%)
	Lower layer of the dermis	20 (17.9%)
	Subcutaneous	9 (8%)
	Rectal mucosa	1 (0.9%)
	Dermis and subcutaneous	1 (0.9%)
**Inflammatory infiltrate**	1+ Low	37 (33%)
	2+ Moderate	67 (60%)
	3+ High	8 (7%)
**Vascular invasion**	No	98 (83.8%)
	Yes	19 (16.2%)
**Perineural invasion**	No	102 (91%)
	Yes	10 (9%)
**FIGO ^1^ stage**	I (A/B) and II	43 (57.4%)
	III A	5 (6.7%)
	III B	19 (25.3%)
	III C	7 (9.3%)
	IV (A/B)	1 (1.3%)
**Relapse**	No	41 (50.6%)
	Yes	40 (49.4%)
**Lymph node metastasis**	No	24 (60%)
	Yes	16 (40%)
**Status**	Alive	33 (38%)
	Death	54 (62%)
**HPV infection**	No	20 (39.2%)
	Yes	31 (60.8%)
**HPV co-infection**	No	20 (64.5%)
	Yes	11 (35.5%)
**HPV type**	High-risk	25 (80.6%)
	Low-risk	3 (9.7%)
	Both	3 (9.7%)

^1^ FIGO: International Federation of Gynecology and Obstetrics.

**Table 2:** Frequency of TSPANs immunohistochemical positive staining (moderate and strong) in VSCC samples (*n* = 117).

Target/ Staining	CD9 *n* (%)	CD63 *n* (%)	CD81 *n* (%)	CD82 *n* (%)	Total *n* (%)	*p*
Negative	66 (59)	18 (17)	84 (80)	56 (52)	223 (52)	<0.0001
Positive	45 (41)	89 (83)	21 (20)	51 (48)	206 (48)	
Total *	111 (100)	107 (100)	105 (100)	107 (100)	429 (100)	

* Total number of analyzed samples; some cores were missed during IHC reactions.

**Table 3:** Correlation analysis of TSPANs.

TSPAN		Correlation Coefficient (r)	*p*
CD9	CD63	0.325	0.001
	CD81	0.423	<0.0001
	CD82	0.597	<0.0001
CD63	CD81	0.202	0.039
	CD82	0.422	<0.0001
CD81	CD82	0.356	<0.0001

**Table 4:** Multivariate proportional hazard analysis of the neoadjuvant chemotherapy status and CD81 protein expression.

Variable	HR (95% CI)	*p*
CD81 expression ^1^	0.41 (0.11–1.49)	0.178
Neoadjuvant chemotherapy ^2^	0.69 (0.19–2.46)	0.576

^1^ Compared to greater expression; ^2^ Compared to treatment. HR, hazard ratio; CI, confidence interval.

**Table 5:** Semi-quantitative measurement of CD63 expression in CD63-siRNA transfected and controls by ICC.

Treatment Group	Non-Metastatic Positive *n* (%)	*n* (%)	Metastatic Positive *n* (%)	*n* (%)	* *p*
Cell Control	2002 (98)	2043 (100)	561 (98)	571 (100)	0.0005
siRNA Control	2318 (98.5)	2354 (100)	603 (98.7)	611 (100)	
CD63 siRNA	410 (21)	1966 (100)	67 (14)	472 (100)	

* Chi-square test.

## Data Availability

All data generated or analyzed during this study are included in this article or Supplementary Material.

## Supplementary Materials

The following are available online at https://www.mdpi.com/article/10.3390/ijms22095015/s1. Figure S1: Tetraspanins protein expression in patient tissues compared to normal tonsil (positive control); Figure S2: Staining pattern for CD9, CD63, CD81, and CD82 in positive control tissues (Tonsils); Figure S3: Specificity of anti-TSPANs by western blot, (a) Detection of CD9, CD63, CD81, CD82, and Actin-B (loading control; C) in total cell lysate from the human breast cancer cell line, MCF7, (b) reactivity of anti-CD63 in total cell lysate from VSCC non-metastatic (SW954) and metastatic (SW962) cell lines. Protein sizes are indicated; Table S1: Comparison between clinical and pathological features from patients, considering neoadjuvant chemotherapy treatment; Table S2: Comparison of HPV types and co-infection in patients with VSCC; Table S3: Prevalence of HPV, considering patients with and without coinfection (n = 31); Table S4: VSCC clinicopathological features and CD9 protein expression; Table S5: VSCC clinicopathological features and CD63 protein expression using two categories (negative and positive expression); Table S6: Comparison between clinical and pathological features and CD63 protein expression using three categories (negative, moderate, and strong expression); Table S7: VSCC clinicopathological features and CD81 protein expression; Table S8: VSCC clinicopathological features and CD82 protein expression.
