# Supplementary material for: Assessment of TSPAN Expression Profile and Their Role in the VSCC Prognosis
Source: Int J Mol Sci. 2021 May 9;22(9):5015. doi: 10.3390/ijms22095015 (PMC8125994; doi:10.3390/ijms22095015)
Supplement: Supplementary file 1 [file ijms-22-05015-s001.zip › ijms-1193583-supplementary.pdf]

Supplementary Materials

Assessment of TSPAN expression profile and their role in the VSCC prognosis

Kelly Pedrozo Ferreira [1], Bruna Cristine de Almeida [1], Laura Gonzalez dos Anjos [1], Glauco Baiocchi [2], Fernando Augusto Soares [3], Rafael Malagoli Rocha [4], Andrey Senos Dobroff [5,6] and Katia Candido Carvalho [1,*]

[1] Laboratório de Ginecologia Estrutural e Molecular (LIM 58), Disciplina de Ginecologia, Departamento de Obstetricia e Ginecologia, Hospital das Clinicas da Faculdade de Medicina da Universidade de Sao Paulo, HCFMUSP, SP, BR Av. Dr Arnaldo 455, sala 4121, Cerqueira Cesar, São Paulo 05403-010, Brazil; keeh.pedroz1@gmail.com (K.P.F.); bruc_10@hotmail.com (B.C.d.A.); lauragonzalezanjos@gmail.com (L.G.d.A.); edmund.baracat@hc.fm.usp.br (E.C.B.)

[2] Department of Gynecology Oncology, A.C.Camargo Cancer Center, Rua Prof Antonio Prudente 211, São Paulo, Brazil; glauco.baiocchi@accamargo.org.br

[3] Department of Pathology, Rede D'OR-São Luiz, Rua das Perobas, 344-Jabaquara, São Paulo 04321-120, Brazil; fasoares@me.com (F.A.S.)

[4] Department of Gynecology, Federal University of São Paulo (UNIFESP), R. Napoleão de Barros, 608, Vila Clementino, São Paulo, SP, 04024-002, Brazil; rafael.malagoli@gmail.com (R.M.R.)

[5] University of New Mexico Comprehensive Cancer Center (UNMCCC), and [6] Division of Molecular Medicine, Department of Internal Medicine, University of New Mexico (UNM) School of Medicine, UNM Health Sciences Center, 1 University of New Mexico, MSC07 4025, Cancer Research Facility (CRF) 321A, Albuquerque, NM 87131, United States of America; adobroff@salud.unm.edu (A.S.D.)

* Correspondence: carvalhokc@gmail.com; Tel.: +55-011-3061-7486 (K.C.C.)

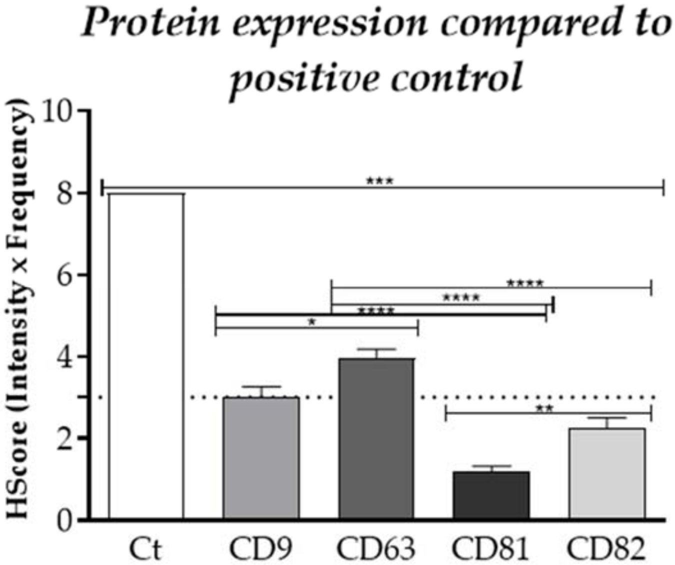

Figure S1. Tetraspanins protein expression in patient tissues compared to normal tonsil (positive control).

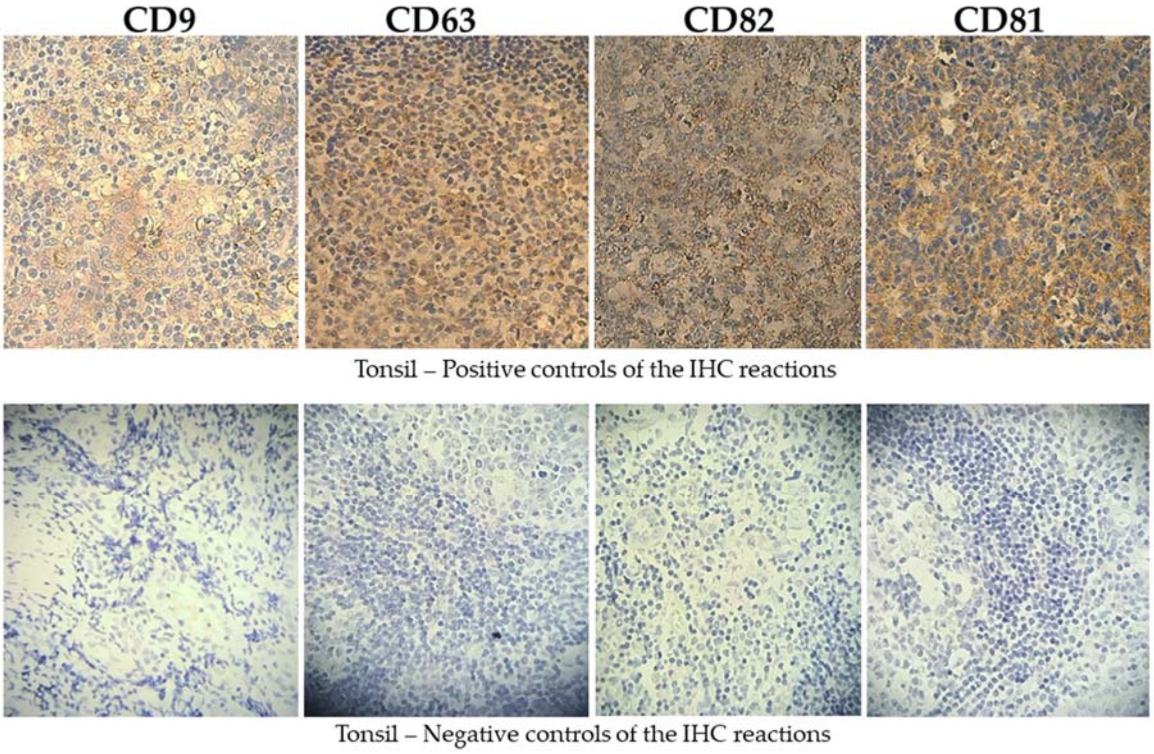

Figure S2. Positive controls for CD9, CD63, CD81 and CD82 using the tissues indicated by antibodies manufacturers´ (Tonsils). Negative controls were obtained using the same tissues and omitting the primaries antibodies, as described in the method section.

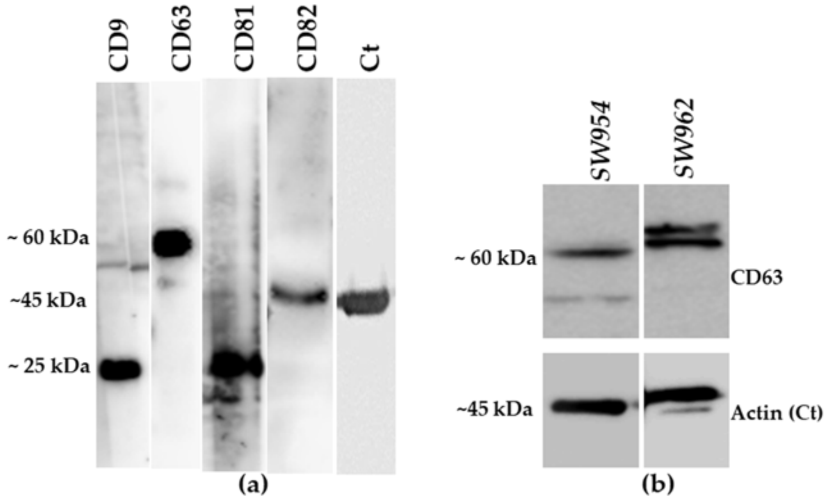

Figure S3. Specificity of anti-TSPANs by Western Blot. **(a)** Detection of CD9, CD63, CD81, CD82, and Actin-B (loading control; C) in total cell lysate from the human breast cancer cell line, MCF7. **(b)** Reactivity of anti-CD63 in total cell lysate from VSCC non-metastatic (SW954) and metastatic (SW962) cell lines. Protein sizes are indicated.

Table S1. Comparison between clinical and pathological features from patients, considering neoadjuvant chemotherapy treatment.

Variables	n	Not treated n (%)	Treated n (%)	Total n (%)	*p*
Labia Majora	77				
No		35 (97)	1 (3)	36 (100)	0.060 [b]
Yes		34 (83)	7 (17)	41 (100)	
Labia Minora	77				
No		27 (96)	1 (4)	28 (100)	0.245 [b]
Yes		42 (86)	7 (14)	49 (100)	
Clitoris	76				
No		47 (94)	3 (6)	50 (100)	0.222 [b]
Yes		22 (85)	4 (15)	26 (100)	
Perineal	78				
No		58 (94)	4 (6)	62 (100)	**0.015** [b]
Yes		11 (69)	5 (31)	16 (100)	
Tumor Size (mm^3)	77				
≤5 mm		10 (67)	5 (33)	15 (100)	**0.014** [a]
6 to 10 mm		13 (93)	1 (7)	14 (100)	
≥10 mm		45 (94)	3 (6)	48 (100)	
Histological diagnosis	79				
VSCC 1		25 (83)	5 (17)	30 (100)	0.765 [a]
VSCC 2		33 (92)	3 (8)	36 (100)	
VSCC 3		6 (86)	1 (14)	7 (100)	
Basaloid carcinoma		5 (83)	1 (17)	6 (100)	
Adjuvant treatment	78				
No		63 (90)	7 (10)	70 (100)	0.229 [b]
Yes		6 (75)	2 (25)	8 (100)	
Lymph node metastasis	38				
No		19 (86)	3 (14)	22 (100)	1.000 [b]
Yes		14 (88)	2 (12)	16 (100)	
Depth of invasion	76				
Superficial dermis		4 (67)	2 (33)	6 (100)	**0.050** [a]
Dermis		40 (91)	4 (9)	44 (100)	
Lower layer of the dermis		13 (87)	2 (13)	15 (100)	
Subcutaneous		9 (90)	1 (10)	10 (100)	
Rectal mucosa		0 (0)	1 (100)	1 (100)	
Inflammatory infiltrate					
1+ Low		18 (86)	3 (14)	21 (100)	0.524 [a]
2+ Moderate		43 (86)	7 (14)	50 (100)	
3+ High		8 (100)	0 (0)	8 (100)	

Vascular invasion					
No		57 (88)	8 (12)	65 (100)	1.000 [b]
Yes		12 (86)	2 (14)	14 (100)	
Perineural invasion					
No		60 (88)	8 (12)	68 (100)	0.608 [b]
Yes		8 (80)	2 (20)	10 (100)	
Clinical FIGO[1] stage	78				
I (A/B) and II		35 (90)	4 (10)	39 (100)	0.974 [a]
III A		6 (86)	1 (14)	7 (100)	
III B		4 (80)	1 (20)	5 (100)	
III C		17 (89)	2 (11)	19 (100)	
IV (A/B)		7 (88)	1 (12)	8 (100)	
Relapse	77				
No		40 (98)	1 (2)	41 (100)	**0.010** [b]
Yes		28 (78)	8 (22)	36 (100)	
Death	77				
No		30 (94)	2 (6)	32 (100)	0.457 [b]
Yes		39 (87)	6 (13)	45 (100)	
HPV infection	41				
No		17 (100)	0 (0)	17 (100)	1.000 [b]
Yes		23 (96)	1 (4)	24 (100)	
HPV coinfection	23				
No		16 (94)	1 (6)	17 (100)	1.000 [b]
Yes		8 (100)	0 (0)	6 (100)	
HPV type	25				
High-risk		21 (100)	0 (0)	21 (100)	**0.021** [a]
Low-risk		2 (67)	1 (33)	3 (100)	
Both		1 (100)	0 (0)	1 (100)	

[a] Chi-square; [b] Fisher exact; [1]FIGO, International Federation of Gynecology and Obstetrics. Bold numbers indicate significant p values.

Table S2. Comparison of HPV types and co-infection in patients with VSCC.

**HPV coinfection**	**High-risk n (%)**	**Low-risk n (%)**	**Both n (%)**	**Total**	***p***
No	18 (90)	2 (10)	0 (0)	20 (100)	
Yes	7 (64)	1 (9)	3 (27)	11 (100)	**0.048**
Total	25 (80.6)	3 (9.7)	3 (9.7)	31 (100)	

Table S3. Prevalence of HPV considering patients with and without coinfection (n = 31).

HPV type	Frequency (%)	HPV Coinfection
High-risk		High-risk
16	17 (58)	16, 18, 33
33	7 (23)	16, 31
53	4 (13)	16, 33
18	2 (6)	16, 33, 82
45	3 (10)	31, 33, 82
82	2 (6)	High-risk and low-risk
31	2 (6)	16, 45, 54
67	1 (3)	16, 11
Low-risk		Low-risk
54	4 (13)	42, 54
11	2 (6)	-
42	1 (3)	-

Table S4. VSCC clinicopathological features and CD9 protein expression.

**Variables**	**n**	**CD9**		**Total**	***p***
		**Negative n (%)**	**Positive n (%)**	**n (%)**	
Age	107				
≤50		7 (58)	5 (42)	12 (100)	1.000 [b]
>50		56 (59)	39 (41)	95 (100)	
Menarche	58				
11 to 14 years old		22 (55)	18 (45)	40 (100)	0.968 [a]
15 to 19 years old		10 (56)	8 (44)	18 (100)	
Menopause	62				
No		1 (33)	2 (67)	3 (100)	0.584 [b]
Yes		33 (56)	26 (44)	59 (100)	
Sexarche	31				
15 to 18 years		6 (37)	10 (63)	16 (100)	0.886 [a]
≥19 years		6 (40)	9 (60)	15 (100)	
Partners	37				
≤2		14 (40)	21 (60)	35 (100)	1.000 [b]
≥3		1 (50)	1 (50)	2 (100)	
Contraceptive	63				
No		32 (55)	26 (45)	58 (100)	0.182 [b]
Yes		1 (20)	4 (80)	5 (100)	
Alcoholism	72				
No		40 (56)	31 (44)	71 (100)	0.444 [b]
Yes		0 (0)	1 (100)	1 (100)	
Smoking	73				

No		32 (57)	24 (43)	56 (100)	0.464 [a]
Yes		8 (47)	9 (53)	17 (100)	
Labia Majora	79				
No		19 (54)	16 (46)	35 (100)	0.526 [a]
Yes		27 (61)	17 (39)	44 (100)	
Labia Minora	80				
No		15 (56)	12 (44)	27 (100)	0.801 [a]
Yes		31 (58)	22 (42)	53 (100)	
Clitoris	78				
No		26 (52)	24 (48)	50 (100)	0.173 [a]
Yes		19 (63)	9 (37)	28 (100)	
Perineal	79				
No		37 (60)	25 (40)	62 (100)	0.173 [a]
Yes		7 (41)	10 (59)	17 (100)	
Tumor Size (mm^3)	106				
≤5 mm		27 (68)	13 (32)	40 (100)	**0.052** [a]
6 to 10 mm		13 (72)	5 (28)	18 (100)	
≥10 mm		22 (46)	26 (54)	48 (100)	
Histological diagnosis	111				
VSCC 1		23 (56)	18 (44)	41 (100)	0.138 [a]
VSCC 2		29 (66)	15 (34)	44 (100)	
VSCC 3		9 (75)	3 (25)	12 (100)	
Basaloid carcinoma		3 (27)	8 (73)	11 (100)	
Verrucous carcinoma		2 (67)	1 (33)	3 (100)	
Neoadjuvant chemotherapy	78				
No		37 (54)	31 (46)	68 (100)	0.732 [b]
Yes		6 (60)	4 (40)	10 (100)	
Adjuvant treatment	81				
No		38 (55)	31 (45)	69 (100)	1.000 [b]
Yes		9 (75)	3 (25)	12 (100)	
Lymph node metastasis	39				
No		13 (57)	10 (43)	23 (100)	0.986 [b]
Yes		9 (56)	7 (44)	16 (100)	
Depth of invasion	106				
Superficial dermis		4 (44)	5 (56)	9 (100)	0.161 [a]
Dermis		43 (64)	24 (36)	67 (100)	
Lower layer of the dermis		12 (63)	7 (37)	19 (100)	
Subcutaneous		3 (30)	7 (70)	10 (100)	
Rectal mucosa		0 (0)	1 (100)	1 (100)	
Inflammatory infiltrate	109				
1+ Low		21 (58)	15 (42)	36 (100)	0.974 [a]

2+ Moderate		38 (58)	27 (42)	65 (100)	
3+ High		5 (62)	3 (38)	8 (100)	
Vascular invasion	111				
No		55 (59)	38 (41)	93 (100)	0.876 [a]
Yes		11 (61)	7 (39)	18 (100)	
Perineural invasion	107				
No		55 (58)	42 (42)	97 (100)	0.514 [b]
Yes		7 (70)	3 (30)	10 (100)	
Clinical FIGO[1] stage	80				
I (A/B) and II		27 (56)	21 (44)	48 (100)	0.996 [a]
III A		3 (60)	2 (40)	5 (100)	
III B		11 (58)	8 (42)	19 (100)	
III C		3 (50)	3 (50)	6 (100)	
IV (A/B)		1 (50)	1 (50)	2 (100)	
Relapse	78				
No		21 (53)	19 (47)	40 (100)	0.474 [a]
Yes		23 (61)	15 (39)	38 (100)	
Death	85				
No		21 (68)	10 (32)	31 (100)	0.133 [a]
Yes		27 (51)	26 (49)	53 (100)	
HPV infection	47				
No		16 (80)	4 (20)	20 (100)	0.337 [b]
Yes		18 (64)	10 (36)	28 (100)	
HPV coinfection	27				
No		14 (74)	5 (26)	19 (100)	0.114 [b]
Yes		4 (40)	6 (60)	10 (100)	
HPV type	27				
High-risk		14 (64)	8 (36)	22 (100)	0.231 [a]
Low-risk		3 (100)	0 (0)	3 (100)	
Both		1 (33)	2 (67)	3 (100)	

[a] Chi-square; [b] Fisher exact; [1]FIGO, International Federation of Gynecology and Obstetrics. Bold numbers indicate significant p values.

Table S5. VSCC clinicopathological features and CD63 protein expression using two categories (Negative and Positive expression).

**Variables**	**n**	**CD63**			
		**Negative n (%)**	**Positive n (%)**	**Total n (%)**	***p***
Age	103				
≤50		2 (17)	10 (83)	12 (100)	1.000 [b]
>50		15 (16)	76 (84)	91 (100)	

Menarche	56				
11 to 14 years old		4 (10)	36 (90)	40 (100)	1.000 [b]
15 to 19 years old		2 (12)	14 (88)	16 (100)	
Menopause	61				
No		0 (0)	3 (100)	3 (100)	1.000 [b]
Yes		7 (12)	51 (88)	58 (100)	
Sexarche	30				
15 to 18 years		2 (13)	13 (87)	15 (100)	0.482 [b]
≥19 years		0 (0)	15 (100)	15 (100)	
Partners	35				
≤2		4 (12)	29 (88)	33 (100)	1.000 [b]
≥3		0 (0)	2 (100)	2 (100)	
Contraceptive	62				
No		7 (12)	50 (88)	57 (100)	1.000 [b]
Yes		0 (0)	5 (100)	5 (100)	
Alcoholism	69				
No		10 (15)	58 (85)	68 (100)	1.000 [b]
Yes		0 (0)	1 (100)	1 (100)	
Smoking	74				
No		8 (15)	45 (85)	53 (100)	0.182 [a]
Yes		6 (29)	15 (71)	21 (100)	
Labia Majora	75				
No		7 (21)	27 (79)	34 (100)	0.209 [a]
Yes		4 (10)	37 (90)	41 (100)	
Labia Minora	76				
No		5 (19)	21 (81)	26 (100)	0.495 [a]
Yes		6 (12)	44 (88)	50 (100)	
Clitoris	74				
No		6 (12)	43 (88)	49 (100)	0.725 [a]
Yes		4 (16)	21 (84)	25 (100)	
Perineal	76				
No		10 (17)	49 (83)	59 (100)	0.438 [b]
Yes		1 (6)	16 (94)	17 (100)	
Tumor Size (mm^3)	102				
≤5 mm		7 (18)	31 (82)	38 (100)	0.191 [a]
6 to 10 mm		5 (29)	12 (71)	17 (100)	
≥10 mm		5 (11)	42 (89)	47 (100)	
Histological diagnosis	107				
VSCC 1		7 (17)	33 (83)	40 (100)	0.316 [a]
VSCC 2		10 (24)	32 (76)	42 (100)	
VSCC 3		1 (8)	11 (92)	12 (100)	

Basaloid carcinoma		0 (0)	10 (100)	10 (100)	
Verrucous carcinoma		0 (0)	3 (100)	3 (100)	
Neoadjuvant Chemotherapy	76				
No		9 (14)	58 (86)	67 (100)	0.610 [b]
Yes		2 (22)	7 (78)	9 (100)	
Adjuvant treatment	18				
No		10 (15)	57 (85)	67 (100)	1.000 [b]
Yes		1 (9)	10 (91)	11 (100)	
Lymph node metastasis	37				
No		6 (26)	17 (74)	23 (100)	0.217 [b]
Yes		1 (7)	13 (93)	14 (100)	
Depth of invasion	102				
Superficial dermis		0	9 (100)	9 (100)	0.169 [a]
Dermis		15 (24)	49 (76)	64 (100)	
Lower layer of the dermis		2 (11)	17 (89)	19 (100)	
Subcutaneous		0 (0)	9 (100)	9 (100)	
Rectal mucosa		0 (0)	1 (100)	1 (100)	
Inflammatory infiltrate	105				
1+ Low		7 (20)	28 (80)	35 (100)	0.515 [a]
2+ Moderate		8 (13)	54 (87)	62 (100)	
3+ High		2 (25)	6 (75)	8 (100)	
Vascular invasion	107				
No		18 (20)	72 (80)	90 (100)	0.070 [b]
Yes		0 (0)	17 (100)	17 (100)	
Perineural invasion	103				
No		17 (18)	77 (82)	94 (100)	1.000 [b]
Yes		1 (11)	8 (89)	9 (100)	
Clinical FIGO[1] stage	77				
I (B / A) and II		10 (21)	37 (79)	47 (100)	0.159 [a]
III A		1 (25)	3 (75)	4 (100)	
III B		0 (0)	18 (100)	18 (100)	
III C		0 (0)	6 (100)	6 (100)	
IV (A / B)		0 (0)	2 (100)	2 (100)	
Relapse	75				
No		8 (20)	32 (80)	40 (100)	0.202 [b]
Yes		3 (9)	32 (91)	35 (100)	
Death	80				
No		6 (19)	25 (81)	31 (100)	0.174 [b]
Yes		4 (8)	45 (92)	49 (100)	
HPV infection	47				
No		4 (21)	15 (79)	19 (100)	1.000 [b]

Yes		6 (21)	22 (79)	28 (100)	
HPV coinfection	29				
No		4 (21)	15 (79)	19 (100)	1.000 [b]
Yes		2 (20)	8 (80)	10 (100)	
HPV type	28				
High-risk		4 (18)	18 (82)	22 (100)	0.100 [a]
Low-risk		2 (67)	1 (33)	3 (100)	
Both		0 (0)	3 (100)	3 (100)	

[a] Chi-square; [b] Fisher exact; [1]FIGO, International Federation of Gynecology and Obstetrics.

Table S6. Comparison between clinical and pathological features and CD63 protein expression using three categories (Negative, Moderate and Strong expression).

**Variables**	**CD63 expression**			**Total n (%)**	***p***
	**Negative n (%)**	**Moderate n (%)**	**Strong n (%)**		
Labia Majora					
No	7 (21)	27 (79)	0 (0)	34 (100)	0.2905 [a]
Yes	4 (10)	36 (88)	1 (2)	41 (100)	
Labia Minora					
No	5 (19)	20 (77)	1 (4)	26 (100)	0.2478 [a]
Yes	6 (12)	44 (88)	0 (0)	50 (100)	
Clitoris					
No	6 (12)	42 (86)	1 (2)	49 (100)	0.7087 [a]
Yes	4 (16)	21 (84)	0 (0)	25 (100)	
Perineal					
No	10 (17)	48 (81)	1 (2)	59 (100)	0.4360 [a]
Yes	1 (6)	16 (94)	0 (0)	17 (100)	
Tumor Size (mm^3)					
≤5 mm	7 (18)	30 (79)	1 (3)	38 (100)	0.2816 [a]
6 to 10 mm	5 (29)	12 (71)	0 (0)	17 (100)	
≥10 mm	5 (11)	42 (89)	0 (0)	47 (100)	
Histological diagnosis					
VSCC 1	7 (17)	33 (83)	0 (0)	40 (100)	**0.0022** [a]
VSCC 2	10 (24)	32 (76)	0 (0)	42 (100)	
VSCC 3	1 (8)	9 (75)	2 (17)	12 (100)	
Basaloid and verrucous carcinoma	0 (0)	13 (100)	0 (0)	13 (100)	
Neoadjuvant treatment					
No	9 (14)	58 (86)	0 (0)	67 (100)	**0.0162** [a]
Yes	2 (22)	6 (67)	1 (11)	9 (100)	
Adjuvant treatment					

No	10 (15)	56 (83)	1 (2)	67 (100)	0.3074 [a]
Yes	1 (9)	9 (82)	1 (9)	11 (100)	
Lymph node metastasis					
No	6 (26)	17 (74)	0 (0)	23 (100)	0.2173 [b]
Yes	1 (7)	13 (93)	0 (0)	14 (100)	
Depth of invasion					
Superficial dermis	0 (0)	9 (100)	0 (0)	9 (100)	0.1343 [a]
Dermis	15 (24)	49 (76)	0 (0)	64 (100)	
Lower layer of the dermis	2 (10)	16 (84)	1 (6)	19 (100)	
Subcutaneous	0 (0)	8 (89)	1 (11)	9 (100)	
Rectal mucosa	0 (0)	1 (100)	0 (0)	1 (100)	
Inflammatory infiltrate					
1+ Low	7 (20)	26 (74)	2 (6)	35 (100)	0.2303 [a]
2+ Moderate	8 (13)	54 (87)	0 (0)	62 (100)	
3+ High	2 (25)	6 (75)	0 (0)	8 (100)	
Vascular invasion					
No	18 (20)	72 (80)	0 (0)	90 (100)	**0.0009** [a]
Yes	0 (0)	15 (88)	2 (12)	17 (100)	
Perineural invasion					
No	17 (18)	76 (81)	1 (1)	94 (100)	0.1050 [a]
Yes	1 (11)	7 (78)	1 (11)	9 (100)	
Clinical FIGO[1] stage					
I (A/B) and II	10 (21)	37 (79)	0 (0)	47 (100)	0.2951 [a]
III A	1 (25)	3 (75)	0 (0)	4 (100)	
III B	0 (0)	17 (94)	1 (6)	18 (100)	
III C	0 (0)	6 (100)	0 (0)	6 (100)	
IV (A/B)	0 (0)	2 (100)	0 (0)	2 (100)	
Relapse					
No	8 (20)	32 (80)	0 (0)	40 (100)	0.2267 [a]
Yes	3 (8)	31 (88)	1 (4)	35 (100)	
Death					
No	6 (19)	25 (81)	0 (0)	31 (100)	0.1939 [a]
Yes	4 (8)	43 (88)	2 (4)	49 (100)	
HPV infection					
No	4 (22)	14 (78)	0 (0)	18 (100)	1.0000 [b]
Yes	6 (21)	23 (79)	0 (0)	29 (100)	
HPV coinfection					
No	4 (21)	15 (79)	0 (0)	19 (100)	1.0000 [b]
Yes	2 (20)	8 (80)	0 (0)	10 (100)	
HPV type					
High-risk	3 (14)	19 (86)	0 (0)	22 (100)	0.0552 [a]

Low-risk	2 (67)	1 (33)	0 (0)	3 (100)
Both	0 (0)	3 (100)	0 (0)	3 (100)

[a] Chi-square; [b] Fisher exact; [1]FIGO, International Federation of Gynecology and Obstetrics. Bold numbers indicate significant p values.

Table S7. VSCC clinicopathological features and CD81 protein expression.

Variables	n	CD81		Total	*p*
		Negative n (%)	Positive n (%)	n (%)	
Age	101				
≤50		9 (82)	2 (18)	11 (100)	1.000 [b]
>50		71 (93)	19 (7)	90 (100)	
Menarche	55				
11 to 14 years old		28 (74)	10 (26)	38 (100)	0.304 [b]
15 to 19 years old		15 (88)	2 (12)	17 (100)	
Menopause	61				
No		2 (100)	0 (0)	2 (100)	1.000 [b]
Yes		45 (76)	13 (24)	59 (100)	
Sexarche	30				
15 to 18 years old		12 (75)	4 (25)	16 (100)	1.000 [b]
≥19 years old		11 (79)	3 (21)	14 (100)	
Partners	36				
≤2		25 (74)	9 (26)	34 (100)	1.000 [b]
≥3		2 (100)	0 (0)	2 (100)	
Contraceptive	61				
No		45 (80)	11 (20)	56 (100)	0.574 [b]
Yes		5 (100)	0 (0)	5 (100)	
Alcoholism	69				
No		57 (84)	11 (16)	68 (100)	1.000 [b]
Yes		1 (100)	0 (0)	1 (100)	
Smoking	72				
No		45 (80)	11 (20)	56 (100)	1.000 [b]
Yes		13 (81)	3 (19)	16 (100)	
Labia Majora	75				
No		32 (94)	2 (6)	34 (100)	**0.029** [b]
Yes		30 (73)	11 (27)	41 (100)	
Labia Minora	76				
No		21 (81)	5 (19)	26 (100)	0.754 [b]
Yes		42 (84)	8 (16)	50 (100)	
Clitoris	74				
No		40 (87)	6 (13)	46 (100)	0.189 [a]

Yes		21 (75)	7 (25)	28 (100)	
Perineal	76				
No		49 (82)	11 (18)	60 (100)	1.000 [b]
Yes		13 (81)	3 (19)	16 (100)	
Tumor Size (mm^3)	100				
≤5 mm		30 (81)	7 (19)	37 (100)	0.479 [a]
6 to 10 mm		16 (89)	2 (11)	18 (100)	
≥10 mm		34 (76)	11 (24)	45 (100)	
Histological diagnosis	105				
VSCC 1		32 (84)	6 (16)	38 (100)	0.900 [a]
VSCC 2		32 (78)	9 (22)	41 (100)	
VSCC 3		9 (75)	3 (25)	12 (100)	
Basaloid carcinoma		9 (82)	2 (18)	11 (100)	
Verrucous carcinoma		2 (67)	1 (33)	3 (100)	
Neoadjuvant Chemotherapy	75				
No		55 (85)	10 (15)	65 (100)	**0.023** [b]
Yes		5 (50)	5 (50)	10 (100)	
Adjuvant treatment	77				
No		54 (82)	12 (18)	66 (100)	0.439 [b]
Yes		8 (73)	3 (27)	11 (100)	
Lymph node metastasis	37				
No		16 (73)	6 (27)	22 (100)	1.000 [b]
Yes		11 (73)	4 (27)	15 (100)	
Depth of invasion	100				
Superficial dermis		7 (88)	1 (12)	8 (100)	0.289 [a]
Dermis		50 (79)	13 (21)	63 (100)	
Lower layer of the dermis		14 (78)	4 (22)	18 (100)	
Subcutaneous		9 (90)	1 (10)	10 (100)	
Rectal mucosa		0 (0)	1 (100)	1 (100)	
Inflammatory infiltrate	103				
1+ Low		26 (84)	5 (16)	31 (100)	0.671 [a]
2+ Moderate		50 (77)	15 (23)	65 (100)	
3+ High		6 (86)	1 (14)	7 (100)	
Vascular invasion	105				
No		70 (80)	18 (20)	88 (100)	1.000 [b]
Yes		14 (82)	3 (18)	17 (100)	
Perineural invasion	101				
No		72 (79)	19 (21)	91 (100)	0.681 [b]
Yes		9 (90)	1 (10)	10 (100)	
Clinical FIGO[1] stage	70				
I (B / A) and II		39 (87)	6 (13)	39 (100)	0.474 [a]

III A		3 (60)	2 (40)	5 (100)	
III B		15 (79)	4 (21)	19 (100)	
III C		4 (67)	2 (33)	6 (100)	
IV (A / B)		1 (100)	0 (0)	1 (100)	
Relapse	74				
No		33 (90)	6 (10)	39 (100)	0.554 [b]
Yes		27 (77)	8 (23)	35 (100)	
Death	80				
No		28 (93)	2 (7)	30 (100)	**0.039** [b]
Yes		37 (74)	13 (26)	50 (100)	
HPV infection	48				
No		17 (85)	3 (15)	20 (100)	1.000 [b]
Yes		24 (86)	4 (14)	28 (100)	
HPV coinfection	29				
No		18 (90)	2 (10)	20 (100)	0.568 [b]
Yes		7 (78)	2 (22)	9 (100)	
HPV type	28				
High-risk		20 (87)	3 (13)	23 (100)	0.534 [a]
Low-risk		2 (67)	1 (33)	3 (100)	
Both		2 (100)	0 (0)	2 (100)	

[a] Chi-square; [b] Fisher exact; [1]FIGO, International Federation of Gynecology and Obstetrics. Bold numbers indicate significant p values.

Table S8. VSCC clinicopathological features and CD82 protein expression.

Variables	n	CD82		Total	*p*
		Negative n (%)	Positive n (%)	n (%)	
Age	102				
≤50		7 (58)	5 (42)	12 (100)	1.000 [b]
>50		48 (53)	42 (47)	90 (100)	
Menarche	56				
11 to 14 years old		23 (67)	16 (33)	39 (100)	0.674 [a]
15 to 19 years old		9 (65)	8 (35)	17 (100)	
Menopause	59				
No		1 (33)	2 (67)	3 (100)	0.568 [b]
Yes		33 (59)	23 (41)	56 (100)	
Sexarche	29				
15 to 18 years old		8 (53)	7 (47)	15 (100)	0.263 [b]
≥19 years old		4 (29)	10 (71)	14 (100)	
Partners	36				
≤2		15 (44)	19 (56)	34 (100)	0.500 [b]

≥3		0 (0)	2 (100)	2 (100)	
Contraceptive	63				
No		32 (55)	26 (45)	58 (100)	0.654 [b]
Yes		2 (40)	3 (60)	5 (100)	
Alcoholism	70				
No		39 (56)	30 (44)	69 (100)	1.000 [b]
Yes		1 (100)	0 (0)	1 (100)	
Smoking	71				
No		30 (56)	24 (44)	54 (100)	0.505 [a]
Yes		11 (65)	6 (35)	17 (100)	
Labia Majora	75				
No		20 (57)	15 (43)	35 (100)	0.975 [a]
Yes		23 (57)	17 (43)	40 (100)	
Labia Minora	77				
No		18 (67)	9 (33)	27 (100)	0.159 [a]
Yes		25 (50)	25 (50)	50 (100)	
Clitoris	74				
No		28 (58)	20 (42)	48 (100)	0.709 [a]
Yes		14 (54)	12 (46)	26 (100)	
Perineal	77				
No		34 (57)	26 (43)	60 (100)	0.874 [a]
Yes		10 (59)	7 (41)	17 (100)	
Tumor Size (mm^3)	102				
≤5 mm		15 (39)	23 (61)	38 (100)	**0.027** [a]
6 to 10 mm		14 (78)	4 (22)	18 (100)	
≥10 mm		24 (52)	22 (48)	46 (100)	
Histological diagnosis	107				
VSCC 1		18 (46)	21 (54)	39 (100)	0.536 [a]
VSCC 2		26 (60)	17 (40)	43 (100)	
VSCC 3		6 (55)	5 (45)	11 (100)	
Basaloid carcinoma		4 (36)	7 (64)	11 (100)	
Verrucous carcinoma		2 (67)	1 (33)	3 (100)	
Neoadjuvant Chemotherapy	76				
No		38 (57)	29 (43)	67 (100)	1.000 [b]
Yes		5 (56)	4 (44)	9 (100)	
Adjuvant treatment	77				
No		41 (61)	26 (39)	67 (100)	**0.004** [b]
Yes		1 (10)	9 (90)	10 (100)	
Lymph node metastasis	37				
No		11 (50)	11 (50)	22 (100)	0.5490 [a]
Yes		9 (60)	6 (40)	15 (100)	

Depth of invasion	102				
Superficial dermis		1 (12)	7 (88)	8 (100)	0.181 [a]
Dermis		35 (54)	30 (46)	65 (100)	
Lower layer of the dermis		9 (50)	9 (50)	18 (100)	
Subcutaneous		6 (60)	4 (40)	10 (100)	
Rectal mucosa		1 (100)	0 (0)	1 (100)	
Inflammatory infiltrate	105				
1+ Low		14 (44)	18 (56)	32 (100)	0.268 [a]
2+ Moderate		38 (58)	27 (42)	65 (100)	
3+ High		3 (47)	5 (63)	8 (100)	
Vascular invasion	107				
No		45 (50)	45 (50)	90 (100)	0.265 [a]
Yes		11 (65)	6 (35)	17 (100)	
Perineural invasion	103				
No		49 (53)	44 (47)	93 (100)	0.747 [b]
Yes		6 (60)	4 (40)	10 (100)	
Clinical FIGO[1] stage	78				
I (B / A) and II		26 (58)	19 (42)	45 (100)	0.743 [a]
III A		2 (50)	2 (50)	4 (100)	
III B		9 (47)	10 (53)	19 (100)	
III C		5 (71)	2 (29)	7 (100)	
IV (A / B)		1 (33)	2 (67)	3 (100)	
Relapse	75				
No		20 (51)	19 (49)	39 (100)	0.391 [a]
Yes		22 (61)	14 (39)	36 (100)	
Death	79				
No		17 (55)	14 (45)	31 (100)	0.901 [a]
Yes		27 (56)	21 (44)	48 (100)	
HPV infection	48				
No		13 (65)	7 (35)	20 (100)	0.959 [a]
Yes		18 (64)	10 (36)	28 (100)	
HPV coinfection	29				
No		15 (79)	4 (21)	19 (100)	0.051 [b]
Yes		4 (40)	6 (60)	10 (100)	
HPV type	29				
High-risk		16 (70)	7 (30)	23 (100)	0.462 [a]
Low-risk		2 (67)	1 (33)	3 (100)	
Both		1 (33)	2 (67)	3 (100)	

[a] Chi-square; [b] Fisher exact; [1]FIGO, International Federation of Gynecology and Obstetrics. Bold numbers indicate significant p values.
